# Hyper-expansion of large DNA segments in the genome of kuruma shrimp, *Marsupenaeus japonicus*

**DOI:** 10.1186/1471-2164-11-141

**Published:** 2010-02-26

**Authors:** Takashi Koyama, Shuichi Asakawa, Takayuki Katagiri, Atsushi Shimizu, Fernand F Fagutao, Rapeepat Mavichak, Mudjekeewis D Santos, Kanako Fuji, Takashi Sakamoto, Toshihide Kitakado, Hidehiro Kondo, Nobuyoshi Shimizu, Takashi Aoki, Ikuo Hirono

**Affiliations:** 1 Graduate School of Marine Science and Technology, Tokyo University of Marine Science and Technology, 4-5-7 Konan, Minato-ku, Tokyo, 108-8477, Japan; 2 Laboratory of Aquatic Molecular Biology and Biotechnology, Graduate School of Agricultural and Life Sciences, the University of Tokyo, Bunkyo-ku, Tokyo 113-8657, Japan; 3 Department of Molecular Biology, Keio University School of Medicine, 35 Shinanomachi, Shinjuku-ku, Tokyo 160-8582, Japan; 4 Advanced Research Center for Genome Super Power, Keio University, 2 Okubo, Tsukuba, Ibaraki, 300-2611, Japan

## Abstract

**Background:**

Higher crustaceans (class Malacostraca) represent the most species-rich and morphologically diverse group of non-insect arthropods and many of its members are commercially important. Although the crustacean DNA sequence information is growing exponentially, little is known about the genome organization of Malacostraca. Here, we constructed a bacterial artificial chromosome (BAC) library and performed BAC-end sequencing to provide genomic information for kuruma shrimp (*Marsupenaeus japonicus*), one of the most widely cultured species among crustaceans, and found the presence of a redundant sequence in the BAC library. We examined the BAC clone that includes the redundant sequence to further analyze its length, copy number and location in the kuruma shrimp genome.

**Results:**

Mj024A04 BAC clone, which includes one redundant sequence, contained 27 putative genes and seemed to display a normal genomic DNA structure. Notably, of the putative genes, 3 genes encode homologous proteins to the inhibitor of apoptosis protein and 7 genes encode homologous proteins to white spot syndrome virus, a virulent pathogen known to affect crustaceans. Colony hybridization and PCR analysis of 381 BAC clones showed that almost half of the BAC clones maintain DNA segments whose sequences are homologous to the representative BAC clone Mj024A04. The Mj024A04 partial sequence was detected multiple times in the kuruma shrimp nuclear genome with a calculated copy number of at least 100. Microsatellites based BAC genotyping clearly showed that Mj024A04 homologous sequences were cloned from at least 48 different chromosomal loci. The absence of micro-syntenic relationships with the available genomic sequences of *Daphnia *and *Drosophila *suggests the uniqueness of these fragments in kuruma shrimp from current arthropod genome sequences.

**Conclusions:**

Our results demonstrate that hyper-expansion of large DNA segments took place in the kuruma shrimp genome. Although we analyzed only a part of the duplicated DNA segments, our result suggested that it is difficult to analyze the shrimp genome following normal analytical methodology. Hence, it is necessary to avoid repetitive sequence (such as segmental duplications) when studying the other unique structures in the shrimp genome.

## Background

The genomes of crustaceans are extremely diverse in their size, with the smallest one having a C-value of 0.14 pg and the largest one weighing 64.62 pg, differing by a factor of 460 [1,2]. Despite their economic importance and production in huge biomass, little is known about the genome organization of crustaceans, especially Malacostraca (including shrimps and crabs) except for the presence of numerous repetitive sequences [3-5]. Recently, although the genomic DNA sequence of a crustacean, water flea *Daphnia pulex*, has been determined, it seems improper to make any conclusion on the crustacean genome because recent phylogenetic analysis based on the DNA sequence data and morphology comparison between Hexapoda (including insects) and Crustacea provided an unexpected finding that Branchiopoda (including the water flea *Daphnia*) is phylogenetically much closer to Hexapoda rather than Malacostraca [6-8]. Therefore, the crustacean genome, in particular the genetic differences between Branchiopoda and Hexapoda group and other sister groups need to be elucidated.

The penaeid shrimp, which is classified into Decapoda in Malacostraca, has been the subject of intense research. Due to its commercial value, several papers on expressed sequence tag (EST) analysis and genetic linkage mapping has been published in the past few years. However, in depth information of their large genome, which is estimated to be about 70% of human genome in size and rich in AT and AAT sequences, is largely unknown [9-11]. As the first step towards understanding the shrimp genome organization, we constructed a BAC library (named MjBL2) from kuruma shrimp (*Marsupenaeus japonicus*) and performed BAC-end sequencing. The results clearly showed extreme redundancy of certain sequences in many BAC clones of the MjBL2 library. We chose one BAC clone (Mj024A04) for detailed analysis in terms of its entire sequence and redundancy in the shrimp genome and found numerous copies of DNA segments that contain the Mj024A04-sequence. This indicates that hyper-expansion of such peculiar DNA segments occurred through segmental duplication events during evolution of the kuruma shrimp genome.

## Results

### BAC library construction and BAC-end sequencing

To provide an overview of the composition and organization of the kuruma shrimp nuclear genome, we constructed BAC library (MjBL2) using kuruma shrimp genomic DNA prepared from hemocytes of 13 shrimps and analyzed the BAC-end sequence (BES). MjBL2 consists of 49,152 BAC clones, which were arrayed in 128 microtiter plates and stored at -80°C. The average insert size was estimated to be 135 kb by *Not*I digestion of 205 randomly selected BAC clones. BES analysis was further performed using 192 BAC clones randomly selected from MjBL2 and retrieved reads were assembled for contiguity [DDBJ: AG993477-AG993734]. Resulting BESs were classified into 29 singletons and 51 contiguous sequences consisting of 2 to 24 reads. Notably, the BLASTN and BLASTX analyses revealed that many of these BESs (20 reads in BLASTN, 55 reads in BLASTX) contained a sequence encoding a protein similar to "inhibitor of apoptosis protein (IAP)" reported in black tiger shrimp (*Penaeus monodon*) (see Additional file 1).

### DNA sequence of a representative BAC clone

**Figure 1:**
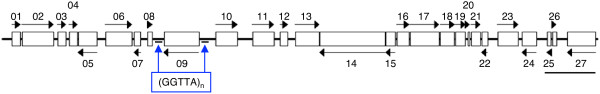
**Schematic organization of putative genes on the kuruma shrimp BAC clone Mj024A04**. Twenty-seven putative genes (boxes) and inter-genic regions (lines) are indicated with transcriptional orientation (arrows). Putative gene 09 is flanked with large GGTTA repeats (double lines).

One of the BAC clones (Mj024A04) that possessed a sequence similar to "black tiger shrimp IAP" gene was randomly selected from MjBL2 for detailed analysis of its entire DNA sequence by employing shotgun sequencing method. The resulting genomic DNA sequence of 120 kb (Mj024A04-sequence) was analyzed by *in silico *annotation, revealing 27 putative genes that apparently seemed to be normal genomic region with exon-intron structure (Figure 1, see Additional file 2 and Additional file 3) [DDBJ: AP010878]. As shown in Figure 1, large GGTTA repeats were found in the middle of the sequence flanking gene 09, which encodes a protein similar to a reverse transcriptase of *Takifugu rubripes *[12]. Notably, of the other 26 genes, three genes (gene 01, 06, and 24) encode a protein homologous to IAP of three species, *Xenopus laevis *(african clawed frog), *Drosophila melanogaster *(fruit fly) and *Rattus norvegicus *(norway rat) and seven genes (gene 11, 13, 14, 15, 16, 17 and 18) were homologous to ORFs in "White Spot Syndrome Virus (WSSV)", the major shrimp pathogenic dsDNA virus, which is highly virulent to penaeid shrimps as well as other crustaceans such as crabs and crayfish [13,14].

### Redundancy of Mj024A04-sequence homologues in the kuruma shrimp BAC library

**Figure 2:**
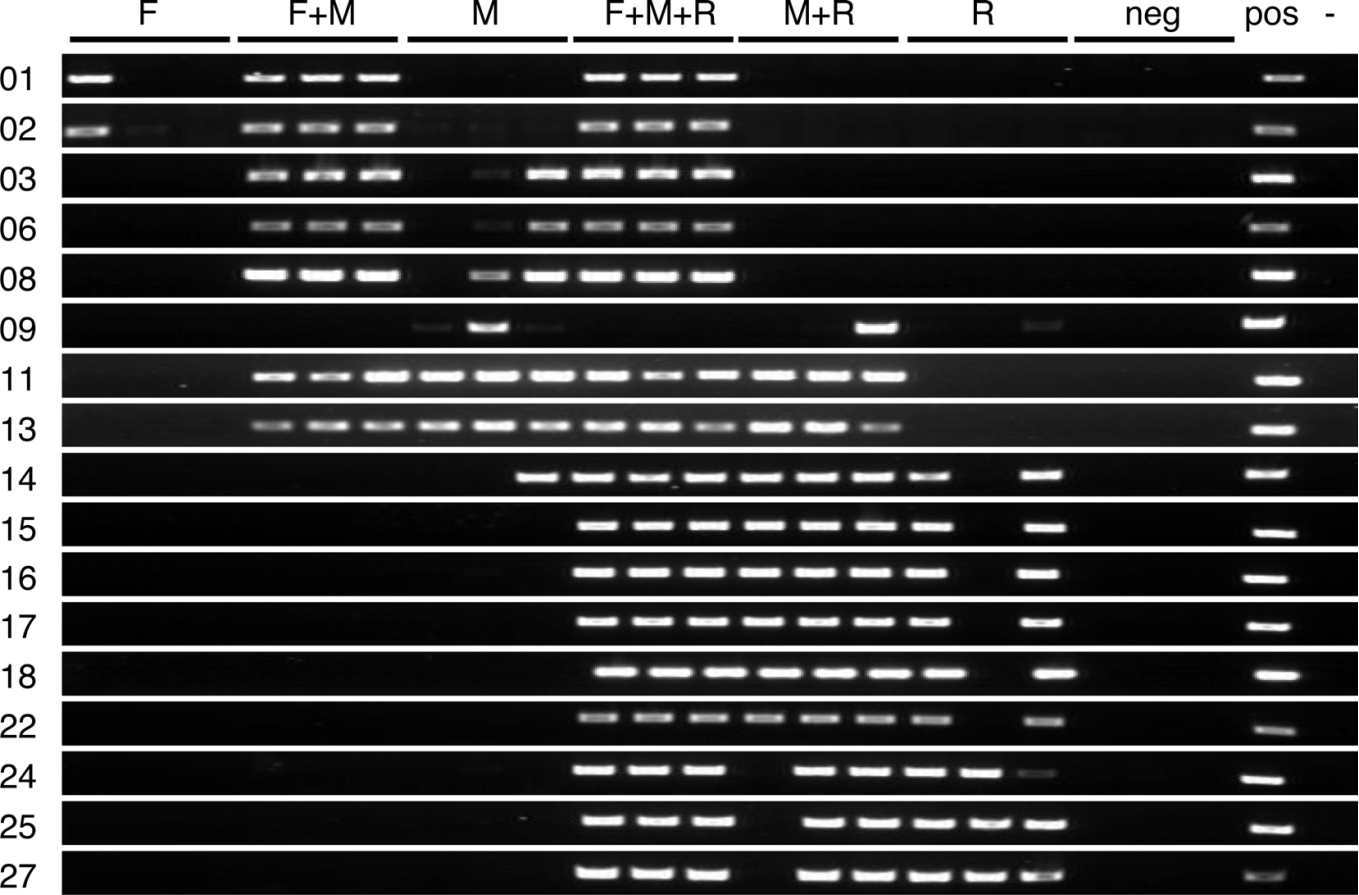
**Amplification of known putative genes using BAC clone samples**. F, M and R probes were designed at the 5'-end, middle and 3'-end portion of the Mj024A04-sequence as described in the result. The putative genes (indicated in left column) were detected with BAC clones that showed different hybridization patterns with F, M and R probes (indicated in top line). Three BAC clones for each hybridization group were tested. Reactions with three BAC clones that showed no signal (negative control: neg), Mj024A04 (positive control: pos) and without templates (-) are also included.

To determine what portion of the Mj024A04-sequence were redundant in the MjBL2-kuruma shrimp BAC library, we performed colony hybridization using three distinct probes that correspond to the 5'-end (F), middle (M) and 3'-end (R) of the Mj024A04-sequence (primers used for probe DNA amplification were shown in Additional file 4). Surprisingly, numerous BAC clones were positive for at least one of the three distinct probes used (200 out of 381: 52.5%; results are shown schematically in Additional file 5) suggesting that relevant DNA fragments are highly redundant in the MjBL2-kuruma shrimp BAC library. The 200 positive BAC clones were sorted into 6 groups based on the hybridization pattern (F, F+M, M, F+M+R, M+R and R) (Additional file 5). Furthermore, we examined possible amplification of the 17 out of 27 putative genes (primers in Additional file 4) on 21 BAC clones (3 clones each from 7 groups) that were proven to be independent by DNA fingerprinting with restriction enzymes *Hin*dIII and *Eco*RI (see Additional file 6). Ten of the 17 genes (gene 01, 02, 03, 06, 08, 09, 22, 24, 25 and 27) were selected because they match other genes in the databases with E-values less than 1e-10 and the other seven (gene 11 and 13 to 18) were selected because of their homology to WSSV genes. As seen in Figure 2, two genes (01 and 02) were present in group F; seven genes (01 to 03, 06, 08, 11 and 13) in group F+M; seven genes (03, 06, 08, 09, 11 13 and 14) in group M; all genes except gene 09 in group F+M+R; twelve genes (09, 11, 13 to 18, 22, 24, 25 and 27) in group M+R; nine genes (14 to 18, 22, 24, 25 and 27) in group R. As expected, some genes were not detected because size of the inserted kuruma shrimp DNA fragments in the selected BAC clones varied. Nevertheless, it is noted that in the group F+M+R, which were supposed to contain all genes, all of the genes except gene 09, which was assigned as retro-transposon, were indeed detected (Figure 2). The independent amplifications of the retro-transposon (gene 09) in the BAC clone samples can be explained by its known nature, which tend to be randomly integrated [15,16]. Random appearance of the retro-transposon suggests that the primordial DNA fragment is void of this gene.

### Detection of Mj024A04-sequence and its copy number in the kuruma shrimp genome

**Figure 3:**
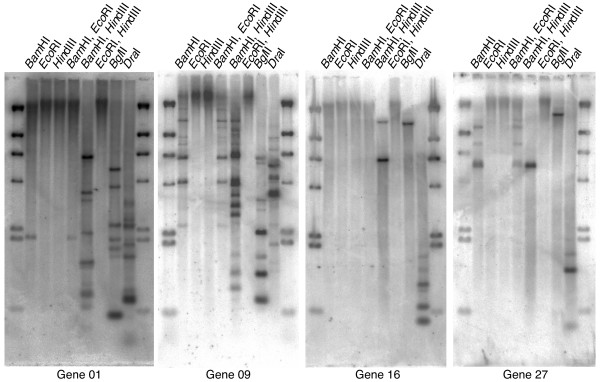
**Southern blot hybridization of kuruma shrimp genomic DNA**. Putative genes (gene 01; *Birc-2 Prov protein*, 09; *Reverse transcriptase*, 16; WSSV-like and 27; *Semaphorin -1A*) used for probe synthesis is indicated at the bottom. Genomic DNA was digested with different combinations of restriction enzymes as indicated at the top.

**Figure 4:**
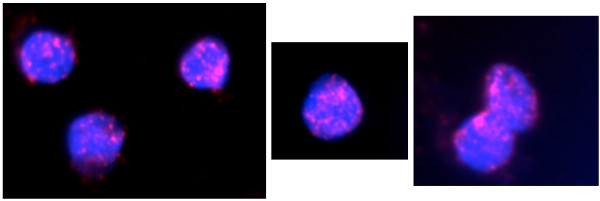
**FISH analysis of Mj024A04-sequence in adult kuruma shrimp testis cells**. Multiple fluorescent signals of Alexa Fluor 594-labeled Mj024A04 are indicated as red spots in the nucleus couterstained with Hoechst 33258 (blue).

**Figure 5:**
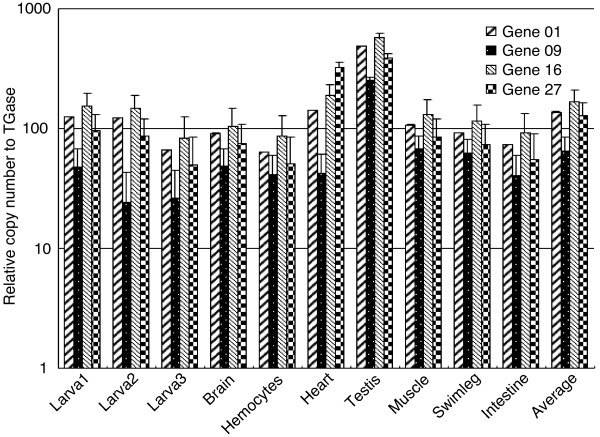
**Copy numbers of 4 putative genes in kuruma shrimp genome**. Putative gene 01 (*Birc-2 Prov protein*), 09 (*Reverse Transcriptase*), 16 (WSSV-like) and 27 (*Semaphorin -1A*) were used to calculate copy numbers in different kuruma shrimp tissues as measured by quantitative PCR. Data represent copy numbers of each gene relative to *TGase *with mean values ± standard deviation (bars) of three experiments.

We next employed Southern blot hybridization to detect multiple copies of Mj024A04-sequence in the kuruma shrimp genome using several different restriction enzymes. Results showed multiple DNA bands for each of the 4 putative genes (gene 01, 09, 16 and 27) (Figure 3), confirming the presence of multiple copies of these genes. In addition, we performed fluorescent *in situ *hybridization (FISH) using labeled Mj024A04 BAC clone. FISH images clearly showed numerous fluorescence spots in the nucleus of the adult shrimp testis cells (Figure 4). With the duplication of the large repeats, we further examined the copy number of the putative genes by quantitative PCR of gene 01, 09, 16 and 27 using genomic DNAs prepared from 7 different organs (brain, hemocytes, heart, testis, muscle, swimleg, and intestine) and 3 larvae. Our results indicated that copy numbers of those putative genes are 100 times more than the putative single copy gene *transglutaminase *(*TGase*), except for the gene 09 (retro-transposon). This suggested the presence of multiple copies of Mj024A04-sequence (Figure 5). Taken all together, our results suggest that large DNA fragment Mj024A04 occurs numerous times in the genome.

### BAC genotyping and PCR detection of putative genes in Mj024A04-sequence

**Figure 6:**
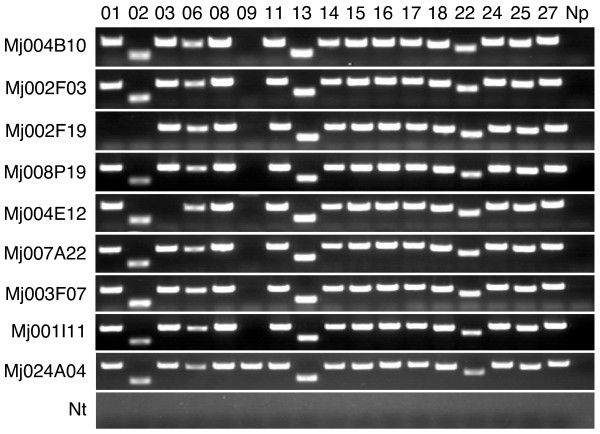
**Amplification of known putative genes using random selected BAC clone samples from different genotypes**. All of BAC clones used in the BAC genotyping were selected based on the hybridization pattern against F, M and R probes that correspond to 5'-end (F), middle (M) and 3'-end (R) of the Mj024A04-sequence as described in the result and method. All genotypes were classified based on 3 distinct microsatellite repeats as described in the result. The putative genes (indicated in top line) were detected with BAC clones that showed different genotypes (indicated in left column). Reactions without templates (Nt) and primers (Np) are included as negative control. Reactions with Mj024A04 as template are also included as positive control.

To exclude a possible cloning bias, we performed BAC genotyping using three microsatellite polymorphisms. 299 different genotypes out of 342 F, M, R positive BAC clones screened from MjBL2 plate 001 to 008 representing 0.2 coverage of shrimp genome were detected (see Additional file 7). PCR-based putative gene detection on eight independent BAC clones selected from different genotypes showed the presence of almost all of the 17 putative genes (Figure 6). Assuming that all shrimps have heterogeneous chromosomes, two genotypes from one allele should be detected. Since we constructed MjBL2 library from 13 individuals, at most 26 different haplotypes were expected for one chromosomal locus. The 299 different genotypes detected indicate that at least 11 different chromosome loci contain duplications of the entire Mj024A04-sequence. As we used only 342 BAC clones from MjBL2 plate 001 to 008, probability estimation method was also performed to estimate how many genotypes could be detected if we performed screening and genotyping with excess BAC clones. This is done with the assumption that all of genotypes were present only once in the 86 diploid chromosomes of the kuruma shrimp [9]. Result indicated 1240 genotypes with 95% confidence interval 960 to 1658, suggesting that at least 48 different chromosome loci might appear in each haploid genome (see Additional file 8).

### Kuruma shrimp Mj024A04-sequence has unique characteristic among arthropod genomes

We performed micro-synteny comparisons of Mj024A04-sequence with genome sequences of other 2 arthropods *Drosophila melanogaster *(version FB2008_02) [17] and *Daphnia pulex *(release 1, 2007/07/07) [18] using TBLASTN algorithm. However, we could not find any micro-synteny relation between Mj024A04 and *Drosophilla *or *Daphnia *genome (data not shown), suggesting the uniqueness of Mj024A04-sequence within known arthropod genomes.

## Discussion

### BAC library construction and BAC-end sequencing for a first characterization of the kuruma shrimp genome

The amount of kuruma shrimp nuclear DNA has been reported to be 2.83 pg indicating that kuruma shrimp genome size is almost the same as other penaeid shrimps such as *Litopenaeus vannamei *and *Penaeus monodon *whose genome size are reported to be approximately 2,000 Mbp [19]. In this study, we first constructed BAC library from the kuruma shrimp. Average insert size of MjBL2 BAC clones were estimated to be 135 kb and total MjBL2 insert size could be calculated as approximately 6,600 Mbp, showing that MjBL2 represented 3.3 times coverage of kuruma shrimp genome. Although MjBL2 is not suitable for physical mapping and genome sequencing because it was constructed from 13 shrimps, MjBL2 is useful as the first step for characterizing the kuruma shrimp genome. We performed BES analysis to acquire the first glimpse into the sequence composition of the unsequenced kuruma shrimp genome. The results of BES analysis were very surprising because even with only 192 clones analyzed, we detected 51 contigs and each contigs contained multiple reads varying from 2 to 24. This suggested that following the typical BAC construction method [20], we obtained multiple copies of the same DNA fragments in the kuruma shrimp genome. However, putative genes such as black tiger shrimp IAP gene homologue that we annotated by BLAST does not seem to be the gene that has potential duplication activity like the transposable elements. To further ascertain the abnormality of the kuruma shrimp genome, we further analyzed these DNA segment in the kuruma shrimp genome.

### Gene contents of a representative BAC clone Mj024A04

Mj024A04 BAC clone randomly selected from BAC clones that possessed black tiger shrimp IAP sequences was fully sequenced and 27 genes were predicted *in silico*. Of the 27 predicted genes, we found three genes homologous to IAP. It is known that apoptosis is a genetically programmed pathway of controlled cell suicide that has critical roles in several processes such as development, tissue homeostasis, DNA damage responses and pathological processes [21]. IAPs have been shown to block apoptosis by inhibition of the proteolytic activity of caspases, the central components of the apoptotic machinery, through direct binding of Baculoviral IAP Repeat (BIR) domains present in the IAPs [22]. Cellular homologues called the BIR-domain-containing protein (BIRPs) are characterized by the presence of a variable number of BIR domains. These homologues have been identified in yeasts, nematodes, flies and higher vertebrates [21-23]. In *Drosophila*, four kinds of IAP homologues (*Thread *or *IAP1*, *IAP2*, *Bruce *and *Deterin *or *CG12265*) have been found [24]. We analyzed the phylogenetic relationships of putative gene 01, 06 and 24 with other BIR domains in several organisms (see Additional file 9). The BIR domains in the putative gene 24 were clustered together with the BIR domains found in black tiger shrimp IAP gene, suggesting the putative gene 24 may have the same function as black tiger shrimp IAP [25]. Particularly of interest, putative gene 06 contains five BIR domains and this is the first report on BIRPs containing more than three BIR domains. BIR domains are known to play important roles in protein-protein interactions and it has been shown that the presence of multiple BIR domains in a single protein molecule increases the affinity of BIRPs to a target protein. In addition, the range of target molecules in which BIRPs can interact also increases with the number of BIR domains [22]. Hence, putative gene 06 that has five BIR domains may be a novel BIRP that has a different function.

Furthermore, of the other 24 genes, we found seven genes homologous to ORFs in WSSV. It is known that certain mammalian dsDNA viruses, such as herpesvirus and poxvirus, mimic structure and function of host genes to evade detection and destruction by the host immune system [26]. Similarly, "potential horizontal gene transfers" has been found in baculoviruses, infectious pathogens of insects [27,28], hence such viral genome structure can be regarded as repositories of important information about host immune processes [29]. The presence of multiple WSSV-like genes in kuruma shrimp genome strongly suggests similar mimicking mechanisms or horizontal gene transfers can also be seen in this virus group. Moreover, with the absence of homologous proteins in the current database, this information will provide a good starting point for understanding unknown WSSV-host interactions.

### The first identification of multiple duplications of large DNA segments in the shrimp genome

As high-resolution whole genome sequences are not yet available for Malacostraca or Decapoda species, it is difficult to make any conclusion if multiple copies of peculiar large DNA segments (Mj024A04-sequence) found in kuruma shrimp are also present in other species. However, micro-synteny analysis revealed that Mj024A04-sequence is not found in two other arthropod genomes, *Drosophila *and *Daphnia*, suggesting that the duplicated large DNA fragments have occurred after establishment of Malacostraca in the Crustacea.

It is also unclear whether the redundancy is the result of polyploidization or segmental duplication. Previous studies revealed a wide range of chromosome numbers and variation of genomic DNA content in several species in Decapoda, suggesting the possibility of polyploidization. However, re-association kinetics of genomic DNA and electrophoretic analysis of enzyme polymorphism have suggested that polyploidization is considered to be a rare event [30,31]. Thus, we assumed that highly redundant large DNA segments in the kuruma shrimp may have arose from segmental duplication events.

Segmental duplications (SDs) are duplicated blocks of genomic DNA, typically ranging in size from 1 kb to 200 kb [32]. SDs are composed of apparently normal genomic DNA containing high-copy repeats and gene sequences with intron-exon architecture, hence it is difficult to detect *a priori *without having well-assigned genome information [32]. In this regard, the human genome is the most studied genome about SDs. Human reference genome contains an abundance of large DNA segments with various copy numbers (from 2 to 18), representing ≥ 5% of the genome, that have been accumulated through evolution over 40 million years [33]. These duplications are shown to be clustered up to 10-fold enrichment within pericentromeric and subtelomeric regions of human chromosomes [32].

SDs are also reported in *Drosophila melanogaster *[34]. In fly, SDs account for ~1.4% of the genome (1.66 Mbp/118.35 Mbp), ranging from 346 bp to 81.1 kb in length. The *Drosophila *genome appears to be significantly poor in large (>10 kb) duplicated blocks with only 7.21% as compared to human genome. The chromosome 4 that appears to be enriched in heterochromatic domains and the pericentromeric regions of the chromosomes X, 2 and 3 in *Drosophila *have also high SD density.

It is reported that subtelomeres are notably rich in degenerate telomeric repeats relative to adjacent single-copy sequences or other genomic regions (~10- and ~100-fold, respectively) in the human genome [33]. We analyzed the number of kuruma shrimp BAC clones harboring GGTTA repeats based on colony hybridization [35]. Results showed that the rate of GGTTA-positive BAC clone are found to be 3 times higher in the BAC clones positive for F, M or R probes than GGTTA-positive rate in all BAC clones tested (45.4% and 17.1%, respectively), suggesting that Mj024A04-sequence and its duplicates are located predominantly in subtelomeric regions and perhaps in pericentromeric regions.

### The absence of transcripts of putative genes in Mj024A04 in several tissues of an adult shrimp

We attempted to detect RNA transcripts for some putative genes analyzed in several tissues of kuruma shrimp but gene expression was so weak despite their high copy number. Together with subtelomeric localization, we considered that this low level of gene expression might be caused by epigenetic control mechanisms, such as CpG-methylation, histone-hypoacetylation and histone-methylation. Although we have attempted to detect CpG-methylation in Mj024A04 segments by genomic Sourthern blot analysis with CpG-methylation insensitive restriction enzyme *Msp*I and its sensitive isoschizomer *Hpa*II, we could not detect any CpG-methylation indicating that transcription level of Mj024A04 is strictly suppressed by other factors (see Additional file 10).

## Conclusions

Genome rearrangements are common phenomena in the eukaryotes, which facilitate not only species diversification but also genetic variation within species. Studies based on the whole genome sequence in primates suggest that significant proportion of the lineage-specific duplication results in different gene expression pattern and mechanistic consequence of changes in the chromosome structure [36]. Furthermore, in a study on *Plasmodium falciparum*, a causative agent of severe human malaria, the authors revealed that eight SDs, which are located on seven different chromosomes, have copy number polymorphism among different strains. The expression levels of the genes found within the SDs are also correlated in part with the gene copy number [37]. These studies strongly suggest that SDs are widely distributed and play significant roles in making biological differences among closely related species. Biological significance of SDs in kuruma shrimp *Marsupenaeus japonicus *is still obscure due to lack of the entire genome sequence information of Decapoda species. Nonetheless, it is interesting how SDs and numerous putative genes such as WSSV homologues act in this species. Furthermore, such hyper-expansion of DNA segments should be taken into serious consideration in whole-genome sequencing and effective construction of genetic linkage maps of this economically important species.

## Methods

### BAC library construction and sequencing of BAC ends

Kuruma shrimp BAC library (MjBL2) was constructed according to the protocol as described previously, with minor modification [20]. Briefly, hemocytes from 13 kuruma shrimps were embedded in 1% low melting agarose plugs and digested in the presence of proteinaseK. Those high molecular weight DNA were partially digested with *Hin*dIII and size fractionated by electrophoresis on CHEF DR-II apparatus (BioRad). Over 150 kb genomic DNA was extracted with NaI and GELase (EPICENTRE), ligated into pBAC-*lac *vector and used for transformation of *E. coli *DH10B T1 phage resistant cells (Invitrogen). A total of 49,152 BAC clones were picked and arrayed on 128 microtiter plates each with 384 wells by Q-Pix (Genetix). High Density Replica (HDR) filters were made using Bio Grid (Bio Robotics). BAC-end sequencing was performed in Dragon Genomics Center (Takara Bio, Shiga, Japan) and retrieved AB1 files were processed for clustering using Phred, Phrap and Consed [38-40]. To identify significant matches to the deposited sequences in the public database, BLASTN and BLASTX algorithms were employed after masking repeat elements with RepeatMasker (version 3.2.8) [41] using cross-match as a search engine.

### Shotgun sequencing, data assembly and analysis

Shotgun library was made from purified DNA of Mj024A04 BAC clone using shotgun library construction kit (Invitrogen). Colony PCR conditions were; an initial denaturation step for 5 min at 95°C, followed by 35 cycles of denaturation step at 95°C for 30 sec, annealing at 55°C for 30 sec and extension step at 72°C for 2 min, and a final extension step at 72°C for 5 min to complete the reaction. M13 forward and reverse primers and rTaq DNA polymerase (Bioneer) mixed in a total volume of 15 *μ*l was used for the colony PCR. Excess primers and dNTPs were removed by ExoSAP-IT (GE Healthcare), following manufacturing instruction. Sequence reactions were performed with SP6 and T7 primers using BigDye Terminator v3.1 Cycle Sequencing Kit (Applied Biosystems) following manufacturing instruction and electrophoresed with ABI 3130xl Genetic Analyzer (Applied Biosystems). Retrieved AB1 files were base-called and assembled by Phred, Phrap and Consed [38-40]. The sequence gaps were closed using a combination of re-sequencing of shotgun clones and BAC direct sequencing. Presumative genes were predicted by GENSCAN [42]. Amino acid sequences of presumed genes were annotated using BLASTP algorithm. Micro-synteny analysis was performed by applying TBLASTN algorithm onto two databases FlyBase (version FB2008_02) [17] and wFleaBase (first release, 2007/07/07) [18].

### Southern blot hybridization analysis

Kuruma shrimp genomic DNA (20 *μ*g) was digested completely with *Bam*HI, *Eco*RI, *Hin*dIII, *Bam*HI and *Eco*RI, *Bam*HI and *Hin*dIII, *Eco*RI and *Hin*dIII, *Bgl*II and *Dra*I, respectively and separated using 0.7% agarose gel. After hydrolysis in 0.25 N HCl and denaturation in 1.5 M NaOH and 0.5 M NaCl, the gel was then blotted onto positive charged nylon membranes (Pall Gelman Laboratory) in 0.4 N NaOH. Hybridization was performed with the probe labelled with [*α*-^32^P]dCTP using Random Primer DNA Labeling Kit Ver. 2 (Takara) at 42°C in PerfectHyb hybridization solution (TOYOBO) for 4 hrs and washing were carried out 3 times with 2× SSC/0.1% SDS at 50°C for 30 min. The autoradiogram was developed with a STARION FLA-9000 Reader (Fujifilm).

### Chromosomal localization of Mj024A04-sequence

Mj024A04 BAC DNA was fluorescent labeled as a FISH probe by nick translation method using the FISH Tag DNA Multicolor kit (Invitrogen) according to manufacture's instructions. The specimens were prepared from the testis cells according to the previous report [43]. After the final heat denaturation of labeled probe and heat denaturation and dehydration of the specimens, hybridization was performed in 2× SSC/65% formamide hybridization buffer at 37°C for 24 hrs. Washings were performed three times with 2× SSC/50% formamide, 1× SSC and 4× SSC/0.1% Tween 20, respectively at 45°C for 5 min. Finally, the specimens were counterstained with Hoechst 33258 (Invitrogen) and examined under a Nikon Eclipse E600 epifluorescence microscope (Nikon). Photographs were taken with a MicroMax Cooled-CCD and IPLab software (Nippon Roper).

### Copy number estimation of Mj024A04 genes

Primer pairs for quantitative PCR were designed for 4 predicted genes (gene 01, 09, 16 and 27) and the putative single copy gene, *transglutaminase *(*TGase*; DQ436474), using Primer Express Software Version 3.0 (Applied Biosystems) (primers were shown in Additional file 4). 0.1 ng of the kuruma shrimp genomic DNA were prepared from the brain, hemocytes, heart, testis, muscle, swimleg, intestine and 3 larvae were used as template in a 20 *μ*l reaction mixture containing 10 *μ*l of SYBR Green PCR Master Mix reagent (Applied Biosystems), 1 *μ*l of genomic DNA template or plasmid containing target DNA sequences as standard, 8.2 *μ*l of deionized water and 0.4 *μ*l of 10 *μ*M forward and reverse primer. PCR reactions were performed and quantified by the 7300 Real-Time PCR System (Applied Biosystems). All of PCR reactions were performed as follows: 50°C for 2 min and 95°C for 10 min, followed by 40 cycles of 95°C for 15 sec and 60°C for 1 min, with a dissociation stage at 95°C for 15 sec, 60°C for 30 sec and 95°C for 15 sec. The PCR reaction was repeated three times for each template. The copy number of each putative gene was estimated by absolute quantification method and Ct values of the amplified target genomic DNA fragments in each sample were computed by the SDS program, using default parameters.

### BAC genotyping using microsatellites

MjBL2 BAC clones showing positive signals against F, M and R probes, which correspond to the 5'-end (F), middle (M) and 3'-end (R) of the Mj024A04-sequence, were used for BAC genotyping with 3 microsatellite markers (MS02, MS33 and MS64; primers used for probe DNA and microsatellite repeats amplification were shown in the Additional file 4). Approximately 15 ng of BAC DNA was used as template in a 10 *μ*l reaction mixture containing 1 *μ*l of 10× Ex Taq buffer, 1 *μ*l of dNTP mixture (2.5 mM each), 0.1 *μ*l of Ex Taq (5 U/*μ*l) (Takara), 1 *μ*l of BAC DNA template, 0.1 *μ*l of 100 *μ*M forward and reverse primer and 6.7 *μ*l of deionized water. PCR reactions were performed by TGradient Thermocycler96 (Biometra) with following condition: first denaturation step at 95°C for 5 min, followed by 40 cycles of 95°C for 30 sec, appropriate annealing temperature for 30 sec and 72°C for 30 sec and a final extension step at 72°C for 5 min. Appropriate annealing temperature determined for each primer pair was 57°C for MS02 and 59°C for MS33 and MS64. Amplified fragments were separated and detected with ABI PRISM 3100 Genetic Analyzer and signal intensity was scored with GeneScan and Genotyper software following instruction manuals (Applied Biosystems). Based on the results obtained from BAC genotyping of 342 BAC clones, we estimated the probable number of different genotypes if more BAC clones (>342) were used for screening and genotyping. The random sampling of size *N *was performed with the assumption that a population having *θ *different genotypes was present with the same abundance. The population was supposed to be large enough so that sampling with replacement is satisfied. Then, let *Y*_*i *_be a random outcome in the *i*-th sampling as follows:

The probability distribution of the initial trial is obviously given as *Pr *(*Y*_1 _= 1) = 1. Furthermore, the conditional distribution of *Y*_*i *_given previous outcomes is expressed by

We now focus on the distribution of an observed number of different genotypes,

The conditional distribution of *K*_*n *_is easily given as

Then, a recursive formula for the marginal distribution of *K*_*n *_can be derived as

once the outcome of *K*_*N *_by the random sampling of size *N *is observed, a likelihood function of *θ *(say *L*(*θ*)) can be obtain through its probability distribution, which is calculated by the recursive formula above. The parameter *θ *is then estimated by maximizing *L*(*θ*). The 100(1-*α*)% confidence interval is also derived by the likelihood profile as , where  is the maximum likelihood estimated of *θ *and *χ*^2^(1-*α*) is the upper 100 *α*-percent of the *χ*^2 ^distribution with the degree of freedom 1.

## Authors' contributions

TKoyama designed and performed all molecular biology experiments and drafted the manuscript. SA and NS assisted in the design of the experiments and helped to draft the manuscript. TKatagiri and RM assisted in the construction of BAC library. FFF assisted in the construction of BAC library and helped to draft the manuscript. AS assisted in the BAC sequence assembly. KF and TS assisted in the BAC genotyping. TKitakado performed statistical analysis of BAC genotyping data. MDS helped to draft the manuscript. HK, TA and IH conceived of the study, participated in its design and coordination and helped to draft the manuscript. All authors read and approved the final manuscript.

## Authors' information

Current affiliation:

MDS:    

1. Vertebrate Section, National Fisheries Research and Development Institute, 940 Quezon Ave., Quezon City, 1103, Philippines                                                                                                                                  

2. Biology Department, Ateneo de Manila University, Katipunan Ave., Loyola Heights, Quezon City, 1108 Philippines

RM: Charoen Pokphand Food Public Company Limited, Shrimp Culture Research Center, 82/2 M 4, Rama II Rd., Bangtorat, Amphor Muang, Samutsakorn City, 74000 Thailand

## Supplementary Material

Additional file 1 **BAC-ends anchored gene homologues identified by BLAST search**. The significant matches (E-value < 0.1) of BAC-End-Sequences against public databases are shown in the list. Click here for file

Additional file 2 **27 putative genes with nearest homologues in the BAC clone Mj024A04**. The BLASTP top hits and the E-values are shown. Click here for file

Additional file 3 **Exon-intron architecture of putative genes in BAC clone Mj024A04**. Exon-intron architectures predicted by GENSCAN are shown. Click here for file

Additional file 4 **Primers used in this study**. The orientation (forward or reverse) of all primers is indicated by 'f' or 'r' in the end of each primer name. Primers whose name is 'Gene--f or r' were used for PCR detection of each putative gene fragment from BAC clones. Primers for quantitative PCR of 4 putative genes (gene 01, 09, 16 and 27) in the BAC clone Mj024A04 and *TGase *for internal control are indicated by 'qt' at the beginning of each primer name. Primers for three microsatellite markers are indicated by 'MS' at the beginning of each primer name. Three microsatellites repeat regions (02, 33 and 64) were determined by RepeatMasker program [41]. Forward primers (f) were labeled by 6-FAM and reverse primers (r) were designed with tailed primer (Applied Biosystems). Click here for file

Additional file 5 **Schematic representation and frequency of BAC clones that hybridized with probe F, M and R**. Number and percentage of positive clones in each group are shown. Data were based on the hybridization results for 381 clones in MjBL2 Plate 24. Location of each probes used in this study is indicated by red boxes. Click here for file

Additional file 6 **DNA fingerprints of kuruma shrimp BAC clones**. 3 BAC clones from each hybridization positive groups (represented by positive probes at the top of each figures) and negative (neg) group were randomly selected. BAC DNA of all clones and Mj024A04 (B) were digested with *Eco*RI and *Hin*dIII. Click here for file

Additional file 7 **BAC genotyping using three microsatellite markers (MS02, 33 and 64) in Mj024A04**. Genotypes representing the same size as the three microsatellite markers were taken as the same group. One base difference was regarded as experimental error. Click here for file

Additional file 8 **Calculation of the number of genotypes in shrimp genomes**. Possible numbers of total genotypes were calculated using recursive formula for the marginal distribution and observed number of different genotypes. X-axis indicates the number of genotypes (*θ*). Y-axis indicates log-likelihood function of each given number of genotype. 90% and 95% confidence intervals (CI) are indicated above. Click here for file

Additional file 9 **Phylogenetic tree of BIR domains of BIRPs**. BIR domains in the putative IAP genes found in Mj024A04 were compared with BIR domains from several organisms. Amino acid sequences of putative IAP gene 01, 06 and 24 were predicted by GENSCAN [42]. Each BIR domains was identified using InterProScan (version 22.0) [44]. Multiple sequence alignment and the phylogenetic tree of BIR domains were constructed using ClustalW after excluding all gap positions and assigning confidence of 1000 bootstrap samples. If multiple BIR domains were observed in a single gene, they are labelled alphabetically at the end of the gene's name. The GenBank identifier (GI) numbers for BIRP amino acid sequences and regions of BIR domains used in the analysis are as follows: bir-1_CAEEL (17564820; 15-88), bir-2_CAEEL (17557418; 22-99 and 165-242), Bir1p_SACCE (6322548; 20-117 and 153-241), bruce_DROME (45550729; 246-322), Bruce_HOMSA (153792694; 284-360), cIAP-1_HOMSA (14770185; 44-115, 182-252 and 267-338), cIAP-2_HOMSA (13639695; 27-98, 167-237 and 253-324), deterin_DROME (21355525; 26-102), gp019_BMNPV (9630835; 27-98 and 129-201), gp041_OPMNV (9629979; 22-93 and 124-195), gp242_MSEV (9631408; 15-77), IAP_GVCP (1170470; 5-75 and 106-177), IAP_PENMO (133754273; 12-83, 103-173 and 253-324), Iap2B_DROME (28573797; 7-78, 111-181 and 210-281), ML-IAP_HOMSA (11545910; 85-156), NAIP_HOMSA (119393878; 58-129, 157-229 and 276-347), OpIAP_ORGPSMNPV (9629973; 16-86 and 109-180), sfIAP_SPOFR (7021325; 98-168 and 208-279), Survivin_HOMSA (59859878; 13-89), survivin_SCHPO (162312092; 20_100 and 115-195), threadB_DROME (24664971; 42-112 and 224-295), VF193_IIV6 (33302608; 35-110), XIAP_HOMSA (12643387; 24-95, 161-232 and 263-332). GeneIDs in which *Daphnia plex *BIRPs were retrieved from wFleaBase [18] and regions of the BIR domains used in the analysis are as follows: Bruce_DAPPL (NCBI_GNO_248214; 320-396), Deterin_DAPPL (NCBI_GNO_774064; 21-105), IAP2_DAPPL (NCBI_GNO_324854; 9-75 and 158-229), thread_DAPPL (NCBI_GNO_284524; 52-122 and 158-229). BIR domains of putative gene 01, 06 and 24 used in the analysis are as follows: Gene 01 (18-95, 124-194 and 244-315), Gene 06 (36-106, 124-199, 268-339, 659-730 and 787-858), Gene 24 (9-80, 100-170 and 248-319). Click here for file

Additional file 10 **Southern blot hybridization of putative genes for detection of CpG-methylation**. Kuruma shrimp genomic DNA (20 *μ*g) was digested completely, electrophoresed and blotted. Hybridization and washing were performed under low stringency condition at 42°C. The restriction enzymes that were used are indicated by their initials (M; *Msp*I, H; *Hpa*II). The putative gene that was used for probe synthesis is indicated at the bottom. Left lane is *λ*/*Hin*dIII marker as size standard. Click here for file
