# Supplementary figures and images for: Hyper-expansion of large DNA segments in the genome of kuruma shrimp, Marsupenaeus japonicus
Source: BMC Genomics. 2010 Feb 26;11:141. doi: 10.1186/1471-2164-11-141 (PMC2838849; doi:10.1186/1471-2164-11-141)

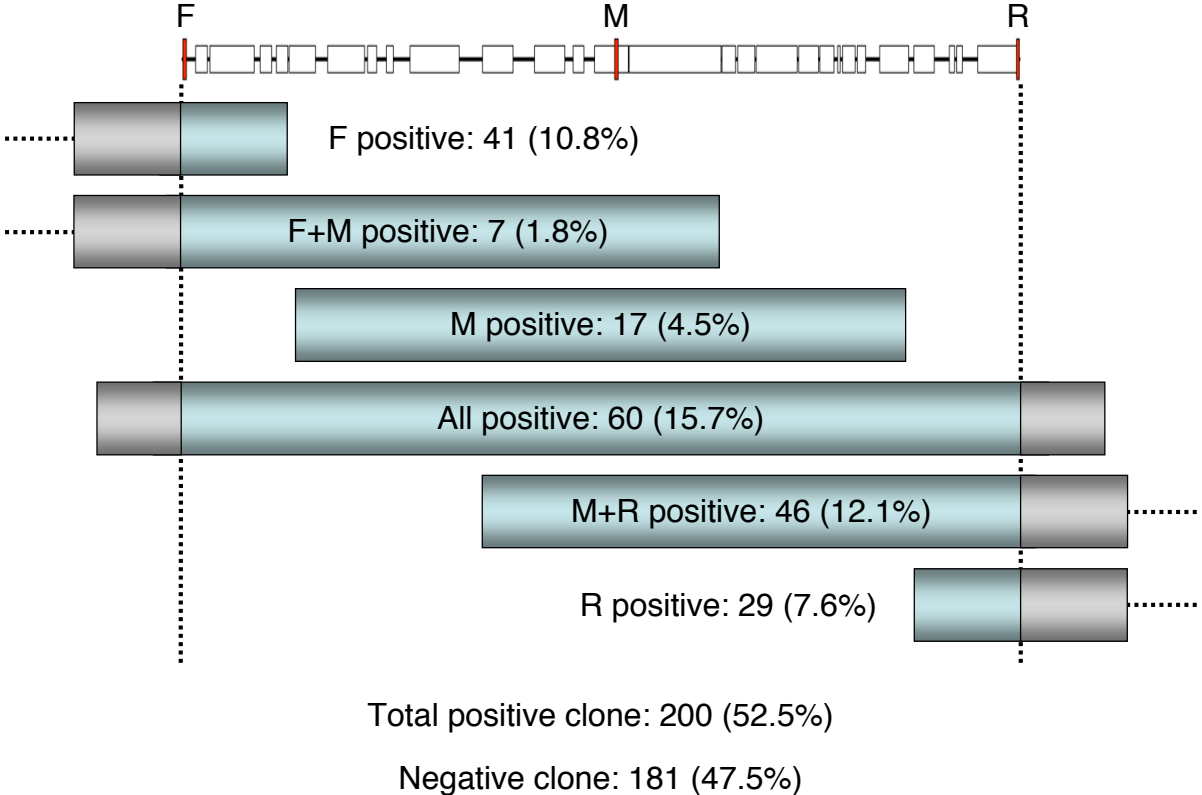

Supplement: Additional file 5 — Schematic representation and frequency of BAC clones that hybridized with probe F, M and R. Number and percentage of positive clones in each group are shown. Data were based on the hybridization results for 381 clones in MjBL2 Plate 24. Location of each probes used in this study is indicated by red boxes. [file 1471-2164-11-141-S5.PDF]

F F+M M F+M+R M+R R neg B

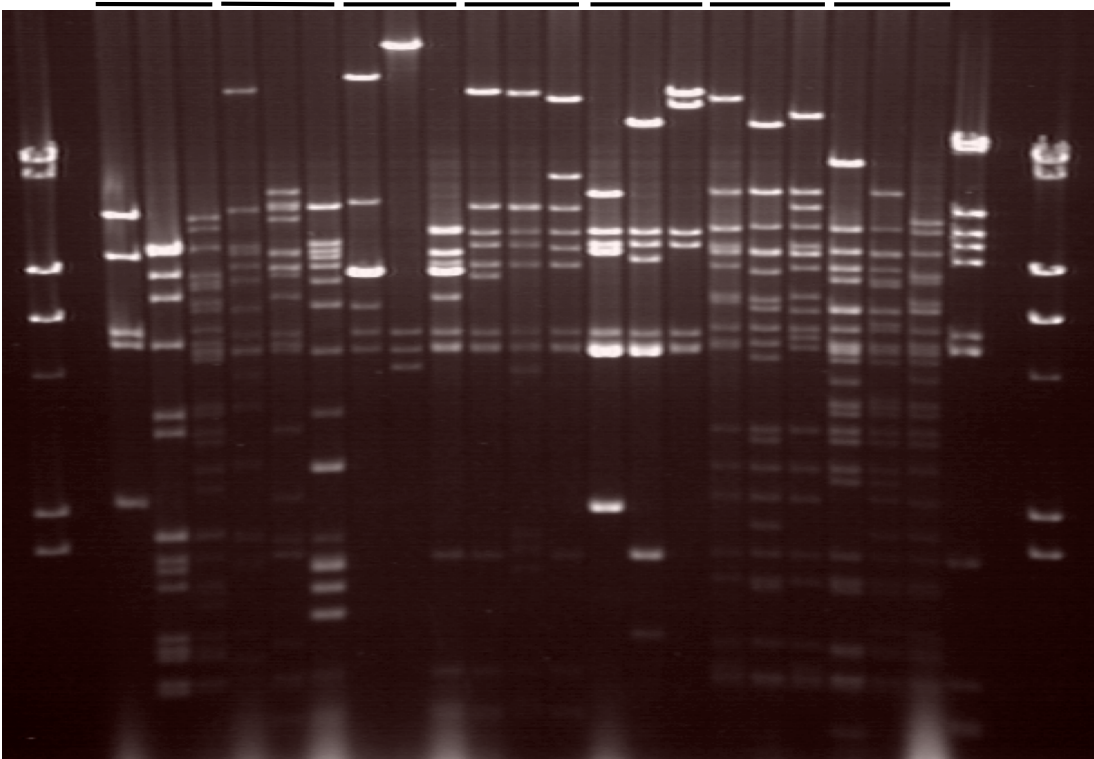

*EcoRI* digestion

F F+M M F+M+R M+R R neg B

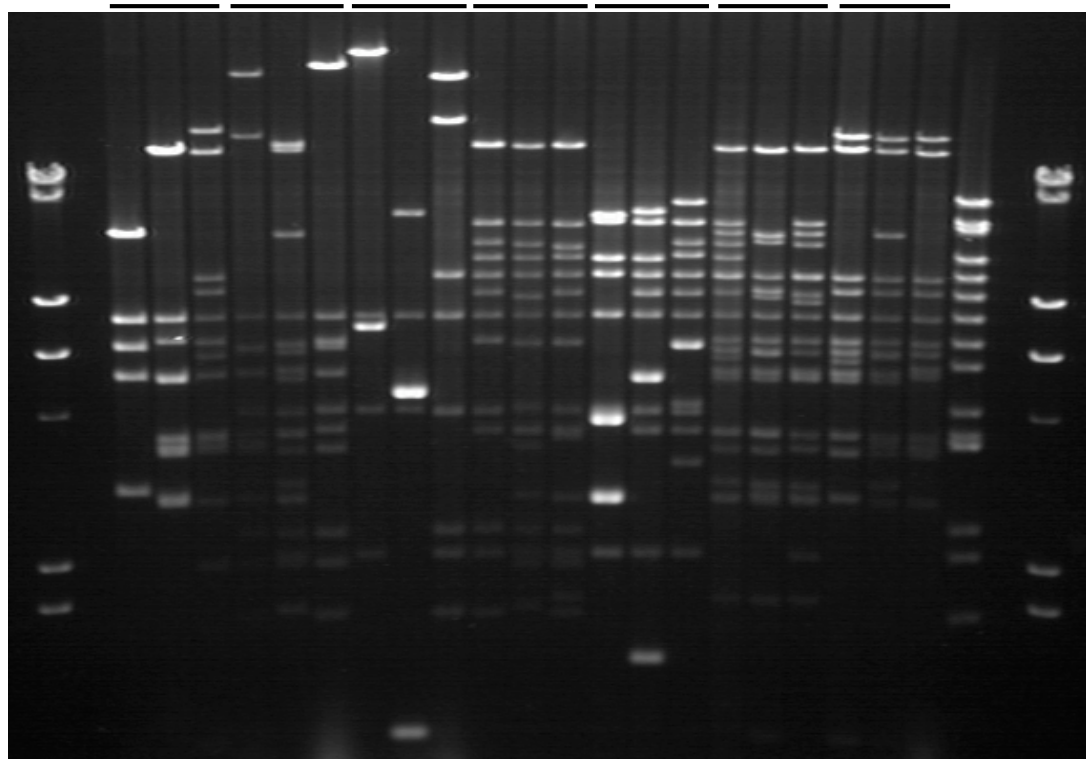

*HindIII* digestion

Supplement: Additional file 6 — DNA fingerprints of kuruma shrimp BAC clones. 3 BAC clones from each hybridization positive groups (represented by positive probes at the top of each figures) and negative (neg) group were randomly selected. BAC DNA of all clones and Mj024A04 (B) were digested with EcoRI and HindIII. [file 1471-2164-11-141-S6.PDF]

Point estimate of theta =1240

90% CI = (998, 1578)

95% CI = (960, 1658)

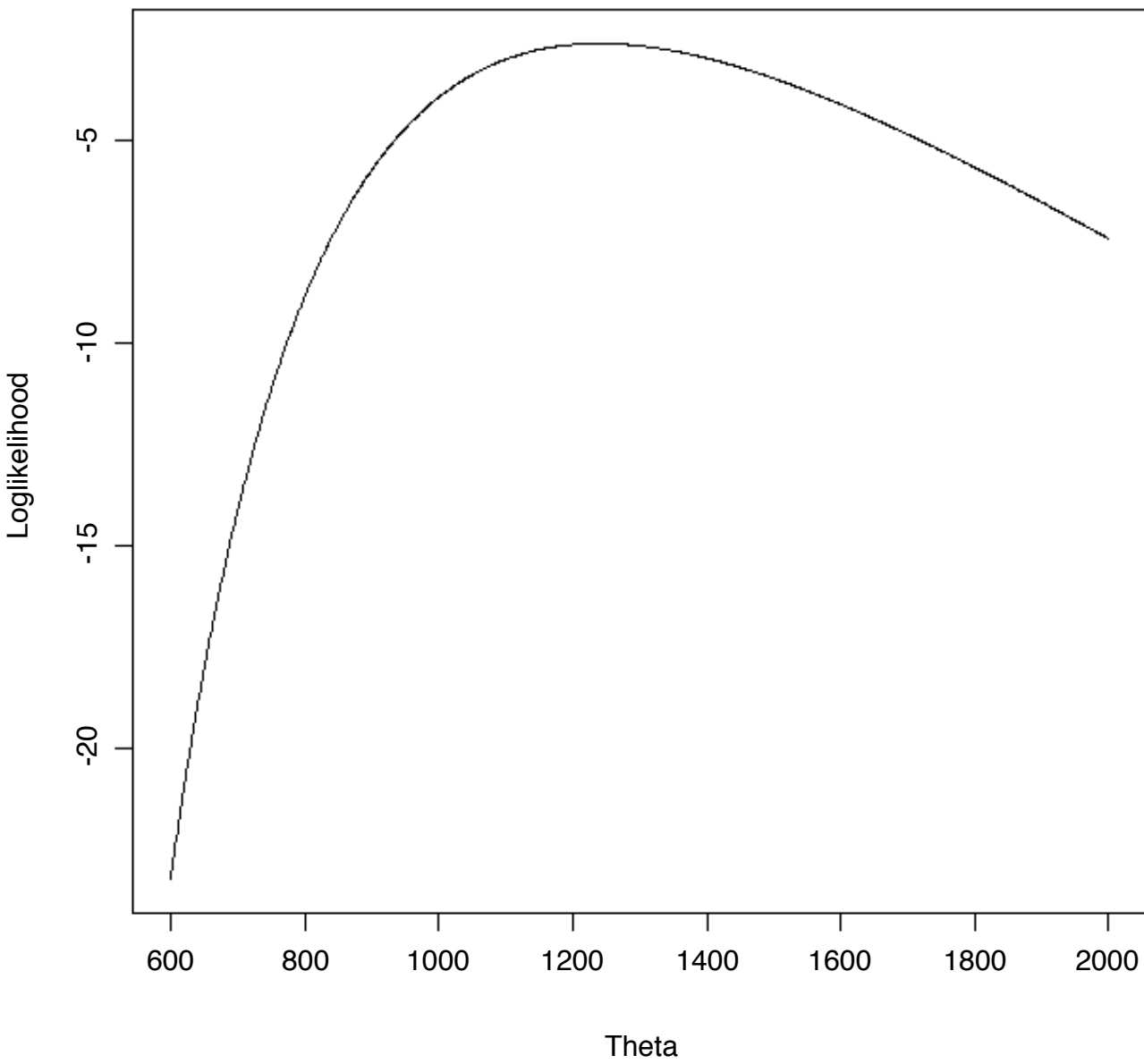

Supplement: Additional file 8 — Calculation of the number of genotypes in shrimp genomes. Possible numbers of total genotypes were calculated using recursive formula for the marginal distribution and observed number of different genotypes. X-axis indicates the number of genotypes (θ). Y-axis indicates log-likelihood function of each given number of genotype. 90% and 95% confidence intervals (CI) are indicated above. [file 1471-2164-11-141-S8.PDF]

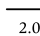

Supplement: Additional file 9 — Phylogenetic tree of BIR domains of BIRPs. BIR domains in the putative IAP genes found in Mj024A04 were compared with BIR domains from several organisms. Amino acid sequences of putative IAP gene 01, 06 and 24 were predicted by GENSCAN [42]. Each BIR domains was identified using InterProScan (version 22.0) [44]. Multiple sequence alignment and the phylogenetic tree of BIR domains were constructed using ClustalW after excluding all gap positions and assigning confidence of 1000 bootstrap samples. If multiple BIR domains were observed in a single gene, they are labelled alphabetically at the end of the gene's name. The GenBank identifier (GI) numbers for BIRP amino acid sequences and regions of BIR domains used in the analysis are as follows: bir-1_CAEEL (17564820; 15-88), bir-2_CAEEL (17557418; 22-99 and 165-242), Bir1p_SACCE (6322548; 20-117 and 153-241), bruce_DROME (45550729; 246-322), Bruce_HOMSA (153792694; 284-360), cIAP-1_HOMSA (14770185; 44-115, 182-252 and 267-338), cIAP-2_HOMSA (13639695; 27-98, 167-237 and 253-324), deterin_DROME (21355525; 26-102), gp019_BMNPV (9630835; 27-98 and 129-201), gp041_OPMNV (9629979; 22-93 and 124-195), gp242_MSEV (9631408; 15-77), IAP_GVCP (1170470; 5-75 and 106-177), IAP_PENMO (133754273; 12-83, 103-173 and 253-324), Iap2B_DROME (28573797; 7-78, 111-181 and 210-281), ML-IAP_HOMSA (11545910; 85-156), NAIP_HOMSA (119393878; 58-129, 157-229 and 276-347), OpIAP_ORGPSMNPV (9629973; 16-86 and 109-180), sfIAP_SPOFR (7021325; 98-168 and 208-279), Survivin_HOMSA (59859878; 13-89), survivin_SCHPO (162312092; 20_100 and 115-195), threadB_DROME (24664971; 42-112 and 224-295), VF193_IIV6 (33302608; 35-110), XIAP_HOMSA (12643387; 24-95, 161-232 and 263-332). GeneIDs in which Daphnia plex BIRPs were retrieved from wFleaBase [18] and regions of the BIR domains used in the analysis are as follows: Bruce_DAPPL (NCBI_GNO_248214; 320-396), Deterin_DAPPL (NCBI_GNO_774064; 21-105), IAP2_DAPPL (NCBI_GNO_324854; 9-75 and 158-229), thread_DAPPL (NCBI [file 1471-2164-11-141-S9.PDF]

*MspI*  
*HpaII*

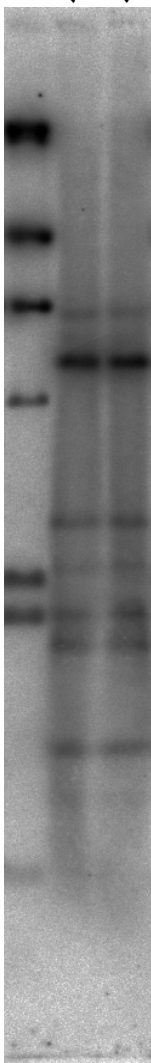

Gene 01

*MspI*  
*HpaII*

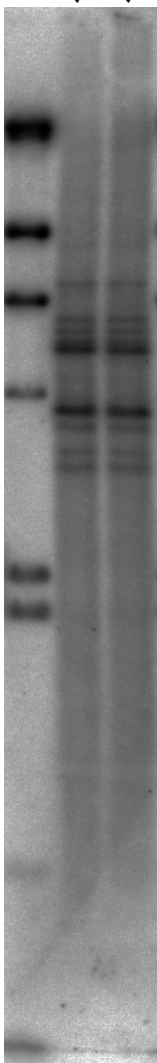

Gene 09

*MspI*  
*HpaII*

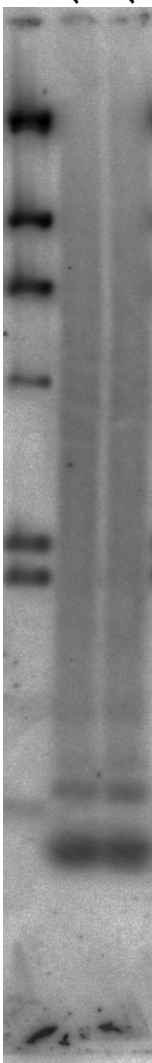

Gene 16

*MspI*  
*HpaII*

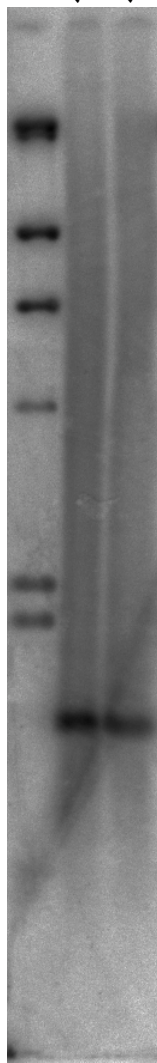

Gene 27

Supplement: Additional file 10 — Southern blot hybridization of putative genes for detection of CpG-methylation. Kuruma shrimp genomic DNA (20 μg) was digested completely, electrophoresed and blotted. Hybridization and washing were performed under low stringency condition at 42°C. The restriction enzymes that were used are indicated by their initials (M; MspI, H; HpaII). The putative gene that was used for probe synthesis is indicated at the bottom. Left lane is λ/HindIII marker as size standard. [file 1471-2164-11-141-S10.PDF]
